# Supplementary material for: Combining structured and unstructured data for predictive models: a deep learning approach
Source: BMC Med Inform Decis Mak. 2020 Oct 29;20:280. doi: 10.1186/s12911-020-01297-6 (PMC7596962; doi:10.1186/s12911-020-01297-6)
Supplement: Supplementary file 1 — Additional file 1. Statistics of the processed MIMIC-III cohort. [file 12911_2020_1297_MOESM1_ESM.docx]

Supplementary material for MIDM-D-20-00161

Table of contents

Figure S1. Length of stay distribution of the processed MIMIC-III cohort.

Table S1. Statistics of collected vital signs and laboratory tests.

Figure S1. Length of stay distribution of the processed MIMIC-III cohort.


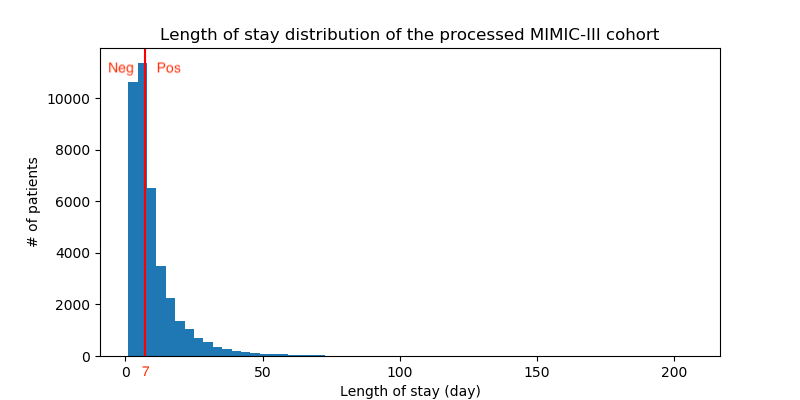


Table S1. Statistics of collected vital signs and laboratory tests.

| Category | Variable | Mean | Std | Min | 10% | 25% | Median | 75% | 90% | Max | Missing rate |
| --- | --- | --- | --- | --- | --- | --- | --- | --- | --- | --- | --- |
| Vital signs | Heart rate | 84.9 | 17.9 | 1 | 63 | 72 | 83.5 | 96 | 96 | 265 | 5.88% |
|  | SysBP | 119.7 | 21.4 | 0.3 | 94.9 | 104 | 117 | 133 | 133 | 311 | 7.99% |
|  | DiasBP | 61.6 | 14.3 | 0.3 | 45 | 52 | 60 | 70 | 70 | 266 | 8.02% |
|  | MeanBP | 78.8 | 15.2 | 1 | 61.5 | 68 | 77 | 87.5 | 87.5 | 298 | 8.17% |
|  | Respiratory rate | 18.8 | 5.3 | 0.2 | 13 | 15 | 18 | 22 | 22 | 69 | 7.60% |
|  | Temperature | 36.9 | 0.9 | 20.9 | 35.9 | 36.39 | 36.89 | 37.44 | 37.4 | 42.1 | 67.62% |
|  | SpO2 | 97.3 | 2.9 | 0.9 | 94 | 96 | 98 | 100 | 100 | 100 | 8.96% |
| Laboratory tests | Anion gap | 14.3 | 3.9 | 1 | 10 | 12 | 14 | 16 | 16 | 59 | 90.31% |
|  | Albumin | 3.2 | 0.7 | 1 | 2.3 | 2.7 | 3.2 | 3.7 | 3.7 | 6.3 | 98.02% |
|  | Bands | 10.2 | 10.4 | 0.9 | 1 | 3 | 7 | 14 | 14 | 79 | 99.58% |
|  | Bicarbonate | 23.6 | 4.9 | 5 | 18 | 21 | 24 | 26 | 26 | 53 | 89.91% |
|  | Bilirubin | 2.1 | 4.6 | 0.1 | 0.3 | 0.4 | 0.7 | 1.7 | 1.7 | 79 | 97.25% |
|  | Creatinine | 1.5 | 1.7 | 0.1 | 0.6 | 0.7 | 1 | 1.5 | 1.5 | 33.2 | 89.72% |
|  | Chloride | 105.4 | 6.6 | 39 | 97 | 102 | 106 | 109 | 109 | 155 | 89.71% |
|  | Glucose | 142.8 | 71.8 | 8 | 87 | 102 | 126 | 160 | 160 | 2286 | 90.32% |
|  | Hematocrit | 31.6 | 5.5 | 5 | 24.9 | 27.7 | 31.1 | 35 | 35 | 71.7 | 87.49% |
|  | Hemoglobin | 10.9 | 2 | 2.8 | 8.4 | 9.5 | 10.7 | 12.1 | 12.1 | 22.1 | 90.51% |
|  | Lactate | 2.7 | 2.2 | 0.2 | 1 | 1.3 | 2 | 3.3 | 3.3 | 32 | 94.18% |
|  | Platelet | 210.3 | 112.6 | 5 | 90.5 | 138 | 194 | 260 | 260 | 2132 | 89.85% |
|  | Potassium | 4.2 | 0.7 | 0.8 | 3.4 | 3.7 | 4.1 | 4.5 | 4.5 | 27.5 | 89.28% |
|  | Ptt | 41.3 | 25.6 | 12.5 | 24.6 | 27.5 | 32.4 | 42.8 | 42.8 | 150 | 92.01% |
|  | Inr | 1.6 | 1 | 0.5 | 1.1 | 1.1 | 1.3 | 1.6 | 1.6 | 38.6 | 92.28% |
|  | Pt | 16.2 | 7.1 | 7.4 | 12.5 | 13.2 | 14.3 | 16.5 | 16.5 | 150 | 92.28% |
|  | Sodium | 138.8 | 5.3 | 82 | 133 | 136 | 139 | 142 | 142 | 182 | 89.82% |
|  | BUN | 26.6 | 22.7 | 1 | 9 | 13 | 19 | 32 | 32 | 270 | 89.77% |
|  | WBC | 12 | 12.3 | 0.1 | 5.2 | 7.4 | 10.3 | 14.2 | 14.2 | 665.6 | 90.52% |
